# Foliar Spray of Cerium Oxide Nanoparticles (CeO_2_ NPs) Improves Lead (Pb) Resistance in Rice

**DOI:** 10.3390/antiox14050552

**Published:** 2025-05-07

**Authors:** Hang Zhou, Junjie Liu, Ziyang Chen, Jing An, Jingxin Huo, Qing Bu, Tao Su, Liming Zhao, Xuefeng Shen, Yingbin Xue, Gangshun Rao, Naijie Feng, Dianfeng Zheng, Rui Zhang

**Affiliations:** 1 College of Coastal Agricultural Sciences, Guangdong Ocean University, Zhanjiang 524088, China; 2 School of Tropical Agriculture and Forestry, Hainan University, Haikou 570100, China; 3 College of Agriculture, South China Agricultural University, Guangzhou 510642, China; 4 South China Branch of National Saline-Alkali Tolerant Rice Technology Innovation Center, Zhanjiang 524088, China

**Keywords:** rice, CeO_2_ NPs, lead stress, transcriptome, metabolome

## Abstract

The widespread use of lead (Pb) has led to serious environmental and human health problems worldwide. The application of oxide nanoparticles (CeO_2_ NPs) in alleviating abiotic stress in plants has received extensive attention. In this study, 50 mg·L^−1^ CeO_2_ NPs can improve Pb resistance and promote rice growth. Specifically, this study observed that CeO_2_ NPs increased the activity of antioxidant enzymes peroxidase (POD), catalase (CAT), and ascorbate peroxidase (APX), but the difference did not reach a significant level. At the same time, CeO_2_ NPs upregulated antioxidant metabolites alpha-linolenic acid, linoleic acid, ferulic acid, and kaempferol under Pb stress. In addition, CeO_2_ NPs upregulated multiple defense response-related genes, such as *OsOPR1* and *OsPR10a*; *RPR10a*, and improved rice carbon flow and energy supply by upregulating sucrose and D-glucose. The results of this study provided technical support for alleviating Pb stress in rice.

## 1. Introduction

Soil can act as a pollution absorber and retain various pollutants, such as heavy metals [1]. Heavy metal pollution in soil systems results from natural processes and human factors [2,3,4] and is widespread worldwide [5]. Over the past 40 years, China’s rapid economic development has negatively impacted the ecological environment, and soil pollution has become increasingly serious [6,7,8]. According to reports, cadmium (Cd), lead (Pb), and arsenic (As) are the most serious pollutants in China’s industrial and agricultural regions in terms of soil pollution and health risks [9,10].

The widespread use of Pb has caused serious environmental and human health problems worldwide, and no known beneficial biological functions of Pb have been found [11,12]. Pb pollution affects seed germination, plant growth, and metabolism [13,14,15]. Plants growing in Pb-contaminated soil absorb Pb through roots and transport it to leaves. Unbound Pb ions in plants become biohazards, directly damaging photosynthetic function [16,17]. High concentrations of Pb may inhibit the enzyme activities associated with chlorophyll biosynthesis, CO_2_ fixation, and the aggregation of the pigment protein complexes in photosystems [18,19]. In addition, plants exposed to Pb stress produce reactive oxygen species (ROS). These ROS deplete plant cell antioxidants and damage cell membrane structure and lipid composition [20]. Pb has been reported to affect the activities of various enzymes in plants. With the increase of Pb^2+^ concentration (5–50 mg·L^−1^), peroxidase (POD) activity and malonaldehyde (MDA) content peaked at 20 mg·L^−1^, while superoxide dismutase (SOD) and catalase (CAT) activities decreased first and then increased in *Potamogeton crispus* [21,22]. These results indicated that plants exposed to Pb stress have complex physiological response characteristics, and the dynamic changes in the activities of different antioxidant enzymes may be closely related to the degree of plant cell damage and stress regulation mechanisms.

The application of nanoparticles in agriculture has been widely reported. Some inorganic forms of nanoparticles have been shown to alleviate plant abiotic stress by enhancing antioxidant defense enzymes [23,24]. Cerium oxide nanoparticles (CeO_2_ NPs) have been reported to exhibit antioxidant behavior and suppress nitric oxide and hydroxyl radicals in biological systems [25]. As a nanozyme, CeO_2_ NPs play an essential role in alleviating abiotic stress factors such as salt, drought, and heavy metals [24]. According to the report, the application of CeO_2_ NPs had an overall positive effect on wheat growth by controlling the transfer of Cd from soil into plant tissues under Cd stress [26]. Therefore, CeO_2_ NPs have great potential as nanozymes to alleviate plant heavy metal stress and improve stress resistance. Up to now, there are no reports on the research of CeO_2_ NPs in alleviating Pb stress in rice.

Based on previous reports, we speculated that applying CeO_2_ NPs may positively regulate Pb resistance in rice. Therefore, this study attempted to verify the hypothesis from multiple perspectives, such as physiology, transcriptomics, and metabolomics, which are conducive to ensuring food security and sustainable agricultural development.

## 2. Materials and Methods

### 2.1. Synthesis of CeO_2_ NPs

CeO_2_ NPs were provided by the College of Agriculture, South China Agricultural University (particle size 5.92 nm; potential −45.07 mV). The synthesis of CeO_2_ NPs was based on the method of An et al. [27].

### 2.2. Experimental Design

This study was conducted in 2024. The test site was an outdoor artificial climate greenhouse at Guangdong Ocean University (natural light, day/night temperatures of 31/25 ± 2 °C, and relative humidity of 60%). The rice test variety was Huanghuazhan, provided by the Physiology and Biochemistry Laboratory of Guangdong Ocean University.

The sterilized rice seeds were soaked in water for 24 h and germinated for 24 h. The germinated seeds were sown in 20.4 cm × 16.9 cm × 14.5 cm (upper diameter/height/lower diameter) sealed-bottom pots, with 57 seeds per pot. Each pot contained 2.5 kg of brick laterite (0.21 g urea, 0.19 g potassium chloride, and 0.25 g diammonium phosphate as base fertilizer). Thirteen days after sowing, 50 mg·L^−1^ CeO_2_ NPs aqueous solution was sprayed on the leaves (4 mL per pot), and water was used as a control. Seven hours later, 583 mg of lead acetate trihydrate was applied to each pot. Twelve days after the first Pb stress treatment, 583 mg of lead acetate trihydrate was added to each pot again (the final concentration of Pb in each pot was 254.79 mg·kg^−1^ (DW)).

In this study, three treatments were set up, namely control (CK); Pb treatment (Pb); and Pb treatment + 50 mg·L^−1^ CeO_2_ NPs (Ce50).

### 2.3. Morphology and Fresh Weight Determination

After the second stress treatment, plant height, shoot fresh weight, root fresh weight, and leaf area were measured on the 4th (first sampling) and 8th (second sampling) days.

The leaf area was measured by a Yaxin-1241 portable leaf area meter (Beijing Yaxin LIYI Technology Co., LTD, China).

### 2.4. Photosynthesis-Related Index Detection

The transpiration rate (Tr), stomatal conductance (Gs), net photosynthetic rate (Pn), and intercellular carbon dioxide concentration (Ci) of the latest fully expanded leaf were measured by LI-6800 portable photosynthetic system (Li-Cor Inc., Lincoln, NE, USA) on the 4th day (first sampling) after the second stress treatment.

### 2.5. Antioxidant Enzyme Activity Detection

The activities of antioxidant enzymes POD, CAT, and ascorbate peroxidase (APX) in leaves were detected on the 4th day (first sampling) after the second stress treatment according to Zhang et al.’s method [28].

### 2.6. Transcriptome Detection

The latest fully expanded leaves were collected for transcriptome sequencing on the 4th day (first sampling) after the second stress treatment. The collected samples were placed in pre-cooled test tubes and temporarily stored in liquid nitrogen. The test tubes containing the samples were then stored in a −80 °C freezer for future use. ShanghaiMajorbio Bio-pharm Biotechnology Co., Ltd. (Shanghai, China) performed RNA purification, reverse transcription, library construction, and sequencing.

### 2.7. Metabolome Detection

The latest fully expanded leaves were collected for metabolome detection on the 4th day (first sampling) after the second stress treatment. The collected samples were placed in pre-cooled test tubes and temporarily stored in liquid nitrogen. The test tubes containing the samples were then stored in a −80 °C freezer for future use. ShanghaiMajorbio Bio-pharm Biotechnology Co., Ltd. (Shanghai, China) performed LC-MS analysis.

### 2.8. Real-Time Quantitative PCR

The latest fully expanded leaves were collected on the 4th day (first sampling) after the second stress treatment. The primers used for RT-qPCR were listed in Table S1. The *OsActin* gene was used as a reference. The relative expression levels were calculated using the 2^−∆∆CT^ method.

### 2.9. Statistical Analysis

SPSS 22 was used to conduct one-way ANOVA. Origin 2021 was used to create figures. This study used Endnote 20 to introduce literature and Grammarly to correct grammatical errors.

## 3. Results

### 3.1. Morphology and Fresh Weight

**Figure 1:**
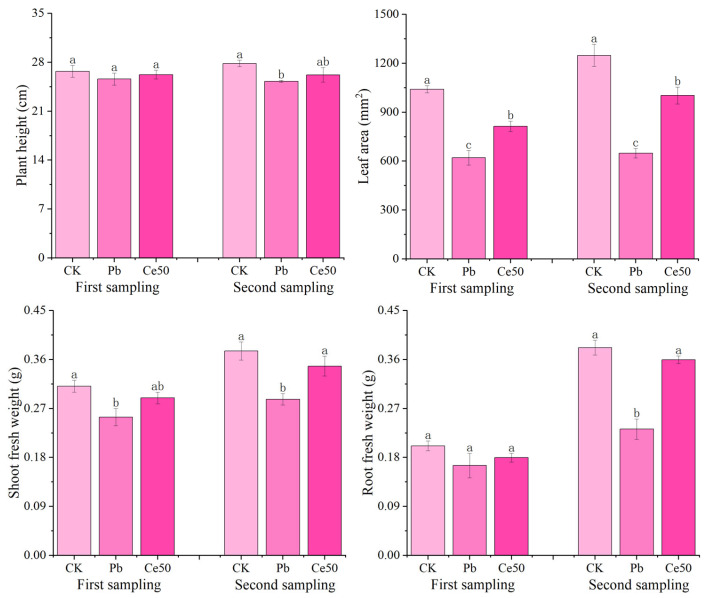
The changes in rice morphology and fresh weight. The different letters indicate significant differences (*p* < 0.05). The value represents the average ± standard error (three replicates).

As shown in Figure 1, there was no significant difference in plant height among the different treatments at the first sampling. The shoot fresh weight and leaf area of CK were significantly higher than those of Pb by 22.44% and 67.85%, respectively; the leaf area of Ce50 was significantly higher than that of Pb by 31.16%, respectively. At the second sampling, the plant height, root fresh weight, shoot fresh weight, and leaf area of CK were significantly higher than those of Pb by 10.16%, 64.51%, 30.89%, and 92.50%, respectively. The root fresh weight, shoot fresh weight, and leaf area of Ce50 were significantly higher than those of Pb by 54.88%, 21.14%, and 54.60%, respectively.

### 3.2. Antioxidant Enzyme Activity and Antioxidant Metabolite Abundance

**Figure 2:**
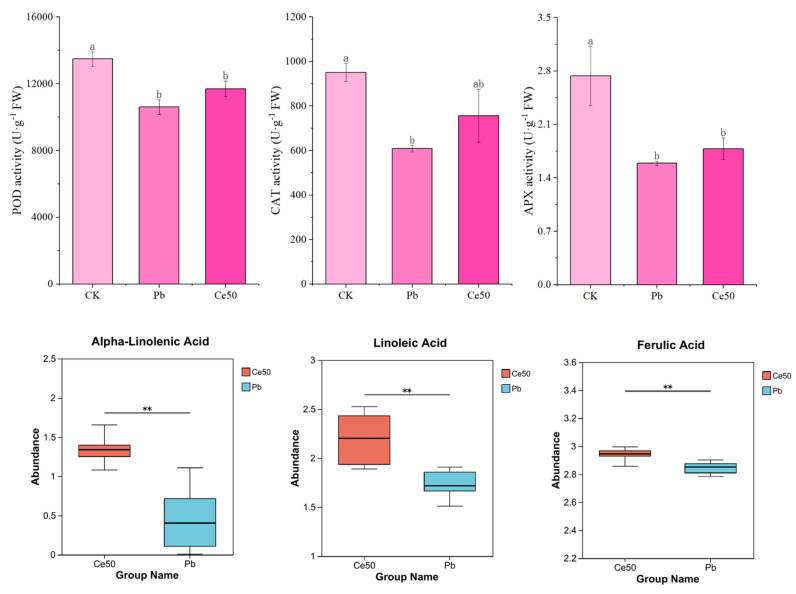
The antioxidant enzyme activity and antioxidant metabolite abundance. For antioxidant enzymes, the different letters indicate significant differences (*p* < 0.05); the value represents the average ± standard error (three replicates). For antioxidant metabolites, ** 0.001 < *p* ≤ 0.01.

As shown in Figure 2, POD, CAT, and APX activities significantly decreased by 27.15%, 56.31%, and 71.88% under Pb stress, respectively, compared with CK. Compared with Pb, the activities of Ce50 of these three enzymes were slightly increased. In addition, this study found that 50 mg·L^−1^ CeO_2_ NPs upregulated alpha-linolenic acid, linoleic acid, and ferulic acid under Pb stress.

### 3.3. Photosynthetic Pigment Content

As shown in Table 1, the chlorophyll a, chlorophyll b, total chlorophyll, and carotenoid contents of Pb at the first sampling were significantly higher than those of CK by 3.24%, 14.07%, 6.35%, and 6.80%, respectively. At the second sampling, the contents of chlorophyll a, chlorophyll b, total chlorophyll, and carotenoids in CK were significantly higher than those of Pb by 4.65%, 21.58%, 9.50%, and 4.12%. The contents of chlorophyll a, chlorophyll b, and total chlorophyll in Ce50 were slightly higher than those in Pb, and the content of carotenoids was significantly higher than that in Pb by 3.47%.

### 3.4. Photosynthesis

**Figure 3:**
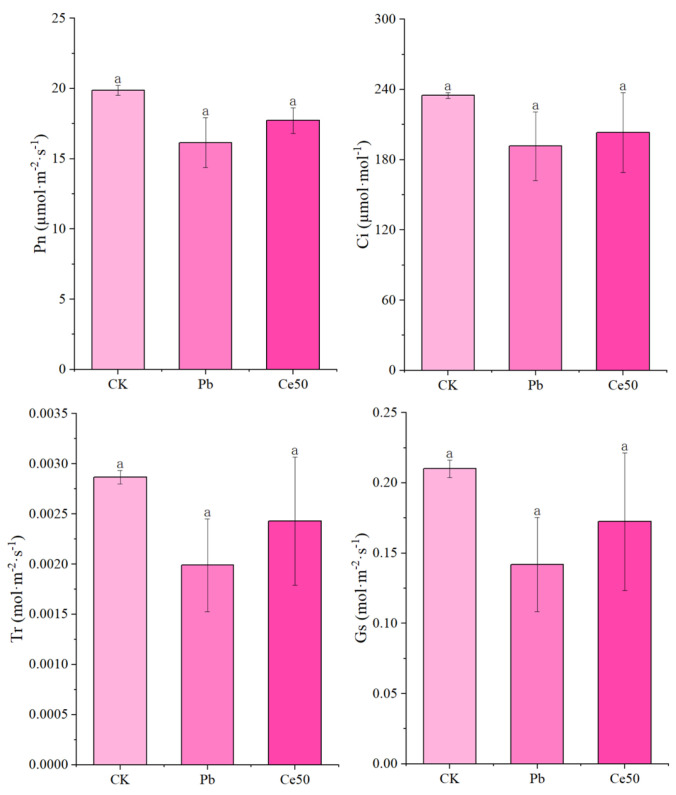
The changes in the photosynthetic parameters of rice. The different letters indicate significant differences (*p* < 0.05). The value represents the average ± standard error (three replicates).

As shown in Figure 3, Pb stress caused a decrease in Pn, Gs, Tr, and Ci, but the differences were not significant. 50 mg·L^−1^ CeO_2_ NPs had a certain effect on improving Pn, Gs, and Tr under Pb stress (not significant).

### 3.5. Transcriptome

#### 3.5.1. Quality Control

This study completed a transcriptome analysis of nine samples. The Q30 base percentage was more than 96.47%. The clean reads of each sample were aligned with the specified reference genome, and the alignment rates ranged from 92.24% to 94% (Tables S2 and S3).

#### 3.5.2. Differentially Expressed Gene (DEG) Statistics

**Figure 4:**
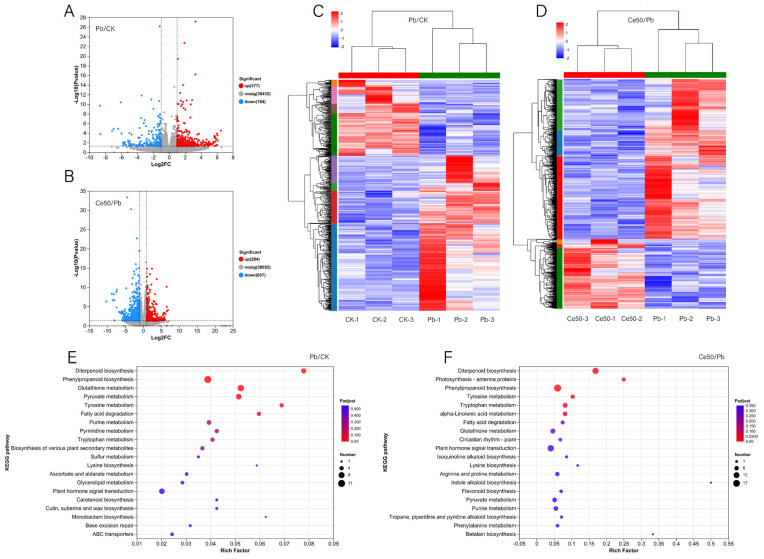
DEGs volcano plot, cluster analysis, and KEGG enrichment analysis. (**A**,**B**) DEGs volcano plot; (**C**,**D**) cluster analysis; (**E**,**F**) KEGG enrichment analysis.

In the Pb/CK comparison group, 377 upregulated DEGs and 184 downregulated DEGs were detected (Figure 4A,C). In the Ce50/Pb comparison group, 284 upregulated and 657 downregulated DEGs were detected (Figure 4B,D).

#### 3.5.3. KEGG Annotation, KEGG Enrichment Analysis, and GO Enrichment Analysis

KEGG annotation analysis found that many DEGs in the Pb/CK comparison group were classified into biosynthesis of other secondary metabolites (17 DEGs), carbohydrate metabolism (15 DEGs), and amino acid metabolism (13 DEGs), indicating that Pb stress may trigger the responses in secondary metabolism and carbohydrate metabolism (Figure S1A). Similarly, in the Ce50/Pb comparison group, most DEGs were attributed to carbohydrate metabolism (34 DEGs), followed by biosynthesis of other secondary metabolites (31 DEGs) and amino acid metabolism (26 DEGs) (Figure S1B).

**Figure 5:**
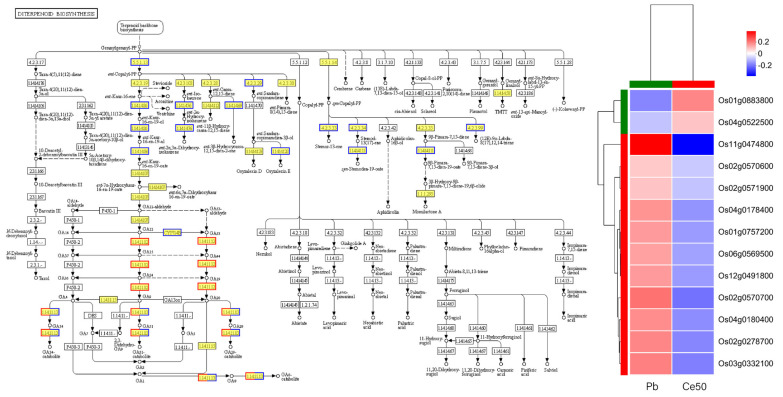
Diterpenoid biosynthesis pathway in the Ce50/Pb comparison group. In the pathway diagram, red represents upregulated genes; blue represents downregulated genes. The copyright of the KEGG map has been obtained.

KEGG enrichment analysis found that the P-adjust of the diterpenoid biosynthesis pathway was the smallest in both comparison groups, reflecting the core role of diterpenoid metabolism in Pb stress response and CeO_2_ NPs may mediate the process of rice encountering Pb stress by regulating diterpenoid metabolism (Figure 4E,F, Figure 5 and Figure S2).

GO enrichment analysis found that 17 DEGs were classified into antioxidant activity (Figure S3B), and 58 DEGs were classified into defense response in the Ce50/Pb comparison group (Figure S3A).

#### 3.5.4. TF Gene Statistics

As shown in Figure S1C, in the Pb/CK comparison group, six genes were classified into the NAC family; five were classified into the WRKY family; the ERF family contained four genes; and MYB also had four TF genes. In the Ce50/Pb comparison group, most of the DEGs were classified into the bHLH family, totaling nine; eight were classified into the WRKY family; eight were classified into the MYB family; the NAC family contained seven; and the HB-other family contained five (Figure S1D).

#### 3.5.5. Expression of Stress Resistance-Related Genes

This study screened some essential genes related to rice stress resistance. These genes were upregulated in Pb + CeO_2_ NP treatment compared with Pb. *OsmiR528* and *OsHKT1;1* are related to rice salt resistance; *OsRCI2-5* and *OsLG3; OsERF62* are related to rice drought resistance; *SOD1-Fe* is a superoxide dismutase gene. In addition, this study selected some DEGs for real-time quantitative PCR detection, and the results obtained were similar to the transcriptome data (Table S4).

### 3.6. Metabolome

#### 3.6.1. Differential Metabolite Statistics, OPLS-DA, and OPLS-DA Permutation Test

**Figure 6:**
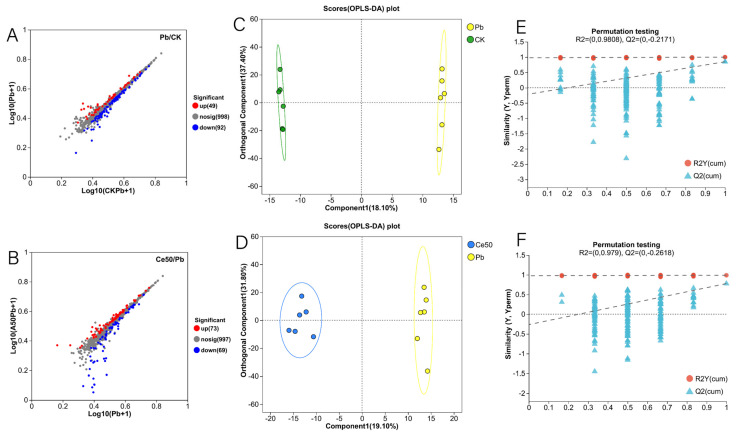
Volcano plot of differential metabolites, OPLS-DA, and OPLS-DA permutation test. (**A**,**B**) Volcano plot of differential metabolites; (**C**,**D**) OPLS-DA; (**E**,**F**) OPLS-DA permutation test ((**E**) in the Pb/CK comparison group; (**F**) in the Ce50/Pb comparison group).

**Figure 7:**
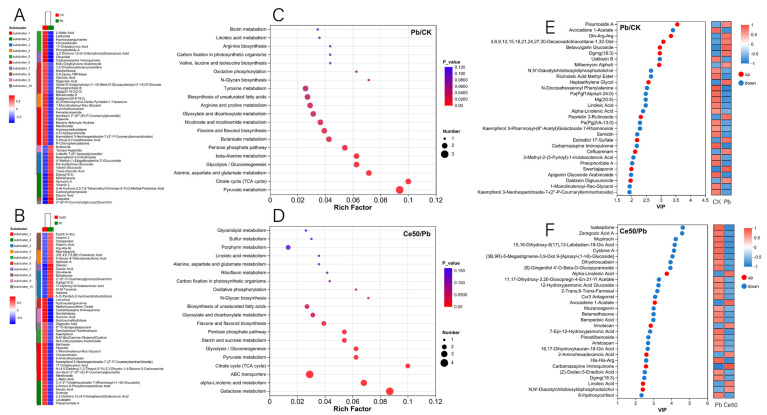
Differential metabolite cluster analysis, KEGG enrichment analysis, and VIP analysis. (**A**,**B**) Differential metabolite cluster analysis; (**C**,**D**) KEGG enrichment analysis; (**E**,**F**) VIP analysis.

In this study, the six samples of each treatment in the OPLS-DA score plot were distributed in the same area, and the different treatments were separated (Figure 6C,D). The OPLS-DA permutation test showed that Q^2^ < 0, indicating that the model was stable and not overfitting (Figure 6E,F). A total of 141 differential metabolites were detected in the Pb/CK comparison group, of which 49 were upregulated, and 92 were downregulated (Figure 6A and Figure 7A). In the Ce50/Pb comparison group, 142 differential metabolites were detected, of which 73 were upregulated, and 69 were downregulated (Figure 6B and Figure 7B).

#### 3.6.2. KEGG Enrichment Analysis

**Figure 8:**
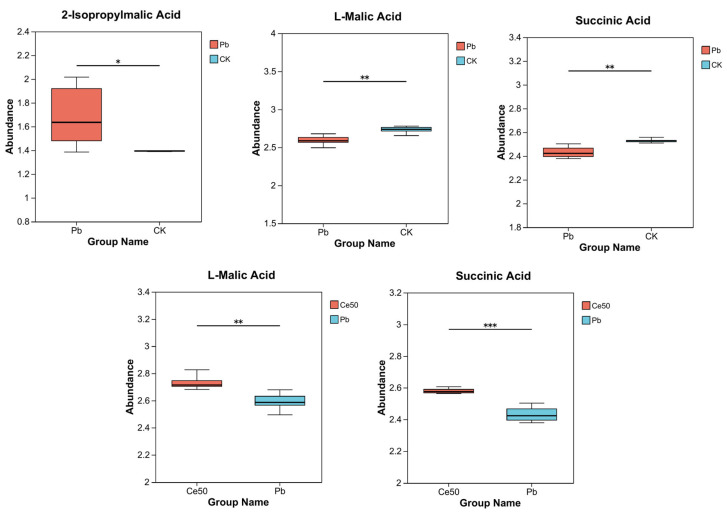
Differential metabolite abundance in the Pb/CK and Ce50/Pb comparison groups. * 0.01 < *p* ≤ 0.05; ** 0.001 < *p* ≤ 0.01; *** *p* ≤ 0.001.

This study conducted KEGG enrichment analysis to study the functions of differential metabolites. The results showed that the P-adjust of the pyruvate metabolism pathway was the smallest in the Pb/CK comparison group. A total of three metabolites were enriched in pyruvate metabolism, namely 2-isopropylmalic acid, L-malic acid, and succinic acid, among which 2-isopropylmalic acid was upregulated, while L-malic acid and succinic acid were downregulated (Figure 7C and Figure 8).

**Figure 9:**
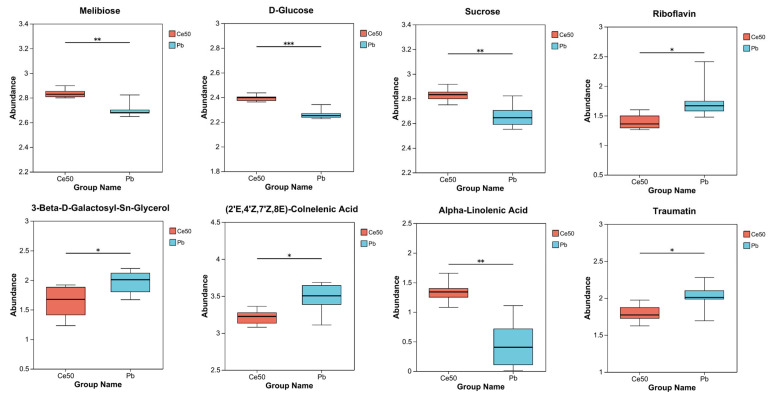
Differential metabolite abundance in the Ce50/Pb comparison groups. * 0.01 < *p* ≤ 0.05; ** 0.001 < *p* ≤ 0.01; *** *p* ≤ 0.001.

The differential metabolites in the Ce50/Pb comparison group were mainly enriched in the galactose metabolism, alpha-linolenic acid metabolism, and ABC transporters pathways. Galactose metabolism was the pathway with the smallest P-adjust in this study. The four differential metabolites enriched in the galactose metabolism pathway were melibiose, D-glucose, sucrose, and 3-beta-D-galactosyl-sn-glycerol; except for 3-beta-D-galactosyl-sn-glycerol, all the others were upregulated. The three differential metabolites enriched in the alpha-linolenic acid metabolism pathway were (2′E, 4′Z, 7′Z, 8E)-colnelenic acid, alpha-linolenic acid, and traumatin; alpha-linolenic acid was upregulated, and other metabolites were downregulated. In addition, four differential metabolites were enriched in the ABC transporters pathway, including upregulated melibiose, D-glucose, sucrose, and downregulated riboflavin (Figure 7D and Figure 9).

#### 3.6.3. VIP Analysis

In the Pb/CK comparison group, the top five differential metabolites in terms of VIP value were pisumoside a, avocadene 1-acetate, gln-arg-arg, 3,6,9,12,15,18,21,24,27,30-decaoxadotriacontane-1,32-diol, and betavulgarin glucoside; except for avocadene 1-acetate, all the others were upregulated (Figure 7E). In the Ce50/Pb comparison group, the top 5 were ixabepilone, zaragozic acid a, mupirocin, 15,16-dihydroxy-8(17),13-labdadien-18-oic acid, and cystone a; all of these metabolites were downregulated (Figure 7F).

#### 3.6.4. KEGG Compound Classification

In the Pb/CK comparison group, a total of four differential metabolites were classified into flavonoids, namely apigenin 7-O-beta-D-rutinoside, tricin, swertiajaponin, and vicenin 2; among them, tricin was downregulated, and the others were upregulated (Figures S4A and S5). In the Ce50/Pb comparison group, three differential metabolites were classified into flavonoids, including silibinin, vicenin 2, and kaempferol; kaempferol was upregulated, while the others were downregulated Figures S4B and S5).

## 4. Discussion

### 4.1. Physiological Responses of Rice to CeO_2_ NPs Under Pb Stress

In this study, Pb stress significantly reduced the shoot fresh weight and leaf area of rice at the first sampling; plant height, shoot fresh weight, root fresh weight, and leaf area all decreased significantly under the influence of Pb stress at the second sampling, indicating that Pb stress had an inhibitory effect on rice growth. Foliar spraying of 50 mg·L^−1^ of CeO_2_ NPs significantly increased the leaf area at the first sampling and significantly increased the shoot fresh weight, root fresh weight, and leaf area at the second sampling, indicating that 50 mg·L^−1^ of CeO_2_ NPs effectively alleviated the adverse effects of Pb toxicity on rice growth.

CeO_2_ NPs have been reported to exhibit antioxidant behavior and suppress nitric oxide and hydroxyl radicals in biological systems [25]. To further explore the mitigation mechanism of CeO_2_ NPs, this study measured the activities of different antioxidant enzymes in leaves and found that Pb stress led to a decrease in POD, CAT, and APX activities. CAT, APX, and POD activities of Ce50 were slightly higher than those of Pb. These three enzymes are mainly responsible for removing ROS. At the same time, 50 mg·L^−1^ CeO_2_ NPs upregulated many antioxidant metabolites, such as alpha-linolenic acid, linoleic acid, and ferulic acid. Alpha-linolenic acid in lipid metabolism is a strong antioxidant and a precursor for synthesizing jasmonic acid, which acts as a signaling molecule to stimulate downstream anti-stress responses [29,30,31]. Linoleic acid has been reported to enhance AsA-GSH cycle efficiency, reduce lipid peroxidation, and decrease ROS accumulation [32]. The antioxidant activity of ferulic acid has been reported to help enhance the plant’s resistance to drought stress [33,34]. These results indicated that foliar spraying of 50 mg·L^−1^ CeO_2_ NPs improved the antioxidant capacity of rice, which helped alleviate the oxidative stress caused by Pb stress.

The detection of photosynthetic pigment contents showed that Pb stress increased photosynthetic pigment-related parameters at the first sampling but reversed at the second sampling. Under Pb stress, the contents of chlorophyll a, chlorophyll b, total chlorophyll, and carotenoids decreased significantly compared with CK at the second sampling. We speculated that this phenomenon was because rice activated the adaptive response mechanism in the initial stress stage, resulting in a short-term increase in photosynthetic pigments. As the stress time continued to increase, the toxic effect of Pb stress began to appear, and then the content of photosynthetic pigments decreased. At the second sampling, foliar spraying of 50 mg·L^−1^ CeO_2_ NPs alleviated the decline of chlorophyll a, chlorophyll b, total chlorophyll content, and the carotenoid content under Pb stress. The changes in the content of photosynthetic pigments indicated that 50 mg·L^−1^ CeO_2_ NPs helped the photosystem to efficiently capture light energy by increasing the pigment content, alleviated the photosynthetic inhibition of rice caused by Pb stress, and enhanced the adaptability to Pb stress.

### 4.2. Response of Rice Metabolism to CeO_2_ NPs Under Pb Stress

This study performed KEGG enrichment analysis on all differential metabolites detected in the Pb/CK comparison group and found that the P-adjust of the pyruvate metabolism pathway was the smallest, indicating that Pb stress may have seriously affected the pyruvate metabolism of rice. A total of three differential metabolites were enriched in the pyruvate metabolism pathway, namely 2-isopropylmalic acid, L-malic acid, and succinic acid. Malic acid increases chlorophyll content, reduces stress damage to photosynthetic structures, and significantly increases plant biomass [35,36]. Succinic acid can be an elicitor candidate and might be used to improve plant stress tolerance [37]. According to reports, succinic acid helps increase resistance to adverse environmental effects. For example, when using a mixture of succinic and lactic acids 45:55 (10 ppm) to treat the root of Asparagus officinal, the seedlings increased their root mass by 40% [38,39]. This study showed that both L-malic acid and succinic acid were downregulated under Pb stress, indicating that Pb stress had a negative impact on the synthesis and accumulation yield of these important metabolites in rice, weakening the stress tolerance. In addition to being enriched in the pyruvate metabolism pathway, they are also essential intermediates in the tricarboxylic acid (TCA) cycle. The TCA cycle was reported to be responsible for driving ATP synthesis and providing carbon skeletons to anabolic processes [40]. Therefore, the downregulation of these metabolites may interfere with the TCA cycle, affecting energy supply and metabolic balance. Interestingly, both metabolites were upregulated in the Ce50/Pb comparison group, indicating that foliar spraying of 50 mg·L^−1^ CeO_2_ NPs under Pb stress could reverse this inhibitory effect.

In the Ce50/Pb comparison group, the differential metabolites were mainly enriched in the galactose metabolism, alpha-linolenic acid metabolism, and ABC transporters pathways. Specifically, sucrose and D-glucose were the two key metabolites enriched in the galactose metabolism pathway in this study. Sucrose is the transportable form of carbon predominantly utilized at the sink to supply the energy required for plant biomass production [41,42] and also to stabilize cellular membranes under stress conditions [43]. Glucose reduces the negative effects of abiotic stress by increasing antioxidant and sugar levels [44]. The upregulation of sucrose and D-glucose in the Ce50/CK comparison group in this study indicated that CeO_2_ NP treatment increased carbon flow and energy supply in rice, which was beneficial to improving rice’s resistance to Pb.

In addition, the KEGG compound classification of the differential metabolites in the two comparison groups revealed that the differential metabolites were mainly classified into flavonoids. In plants, flavonoids play a role in protecting against biotic and abiotic stresses [45,46]. Flavonoids improve plants’ tolerance to abiotic stress by improving antioxidant capacity [47]. In this study, foliar spraying of 50 mg·L^−1^ CeO_2_ NPs upregulated kaempferol, which functions to protect plants from oxidative stress [48], verifying that CeO_2_ NPs have the effect of improving antioxidant capacity.

### 4.3. Response of Rice Gene Expression to CeO_2_ NPs Under Pb Stress

Plant defense priming is a physiological process by which a plant is prepared to respond more quickly or aggressively to future biotic or abiotic stress [49,50,51]. KEGG enrichment analysis found that the diterpenoid biosynthesis in the Pb/CK and Ce50/Pb comparison groups was the most significant pathway, reflecting the core role of diterpenoid metabolism in Pb stress response and that CeO_2_ NPs may mediate the adaptability to Pb stress by regulating the diterpenoid metabolism pathway.

GO enrichment analysis showed that 17 DEGs were classified into antioxidant activity in the Ce50/Pb comparison group. Among these DEGs, *SOD1-Fe* (Os06g0115400) was significantly upregulated. This is a superoxide dismutase gene involved in the antioxidant response of rice.

Furthermore, this study found that 58 DEGs were classified into defense response in the Ce50/Pb comparison group, which meant that foliar spraying of CeO_2_ NPs may initiate the defense mechanism of rice against Pb stress by regulating the expression of these genes. *OsOPR1* (Os06g0216300) is a cadmium stress-responsive gene [52] upregulated in the Ce50/Pb comparison group. Wu et al. reported that overexpression of *OsOPR1* improved the cadmium tolerance of yeast cells by affecting the expression of antioxidant enzyme-related genes and reducing the cadmium content in yeast cells [52]. Moreover, *OsOPR1* encodes 12-oxo-phytodienoic acid (OPDA) reductase, an enzyme involved in jasmonic acid biosynthesis [53]. *OsPR10a; RPR10a* (Os12g0555500) was another gene focused on in this study, and it was upregulated after using CeO_2_ NPs under Pb stress. According to the previous report, the overexpression of *OsPR10a* in rice can significantly enhance resistance to infection by the pathogen *Xoo* and *Xanthomona campestris* pv. *campestris (Xcc)*, respectively [54]. Jasmonic acid has a strong induction effect on *RPR10a* [55]. Therefore, the simultaneous upregulation of *OsOPR1* and *OsPR10a* indicated that foliar spraying of CeO_2_ NPs might activate the jasmonic acid signaling pathway, thereby improving the ability of rice to cope with Pb stress.

This study also screened some genes related to rice stress resistance, which were upregulated after CeO_2_ NP treatment, such as *OsmiR528*, *OsLG3*; *OsERF62*, *OsRCI2-5*, and *OsHKT1*; *1*. *OsMIR528* was reported to be a positive regulator in salt stress [56]. *oshkt1*;*1* mutant plants show hypersensitivity to salt stress [57]. The upregulation of salt response-related genes suggested that CeO_2_ NPs may have the potential to provide salt tolerance to rice, which is consistent with the results of Zhou et al. [58]. Meanwhile, *OsRCI2-5* was reported to be a drought-resistant gene [59]. The overexpression of *OsLG3* significantly improves drought tolerance in rice [60]. These revealed that CeO_2_ NPs may potentially mediate drought resistance in rice.

In addition, based on the statistical results of TF families, this study found that bHLH, WRKY, MYB, and NAC families contained most TF genes in the Ce50/Pb comparison group. These TF families play a role in plant adaptive responses to abiotic stresses [61,62,63,64]. Many DEGs were classified into these families, suggesting that CeO_2_ NPs may mediate plant stress resistance by regulating the expression of these TF genes. However, these four families contain a large number of DEGs, and these DEGs may be involved in a complex gene regulatory network. Further studies on these transcription factors’ interactions and downstream target genes will help reveal more details of the regulation of CeO_2_ NPs on rice under Pb stress.

## 5. Conclusions

This study preliminarily explored the positive regulatory role of CeO_2_ NPs in alleviating Pb stress in rice, especially in improving antioxidant capacity, which is beneficial to alleviate rice oxidative stress under Pb stress and is one of the characteristics of the regulatory mechanism of CeO_2_ NPs in this study. In addition, CeO_2_ NP treatment increased carbon flow and energy supply. KEGG enrichment analysis of DEGs revealed the core role of diterpenoid metabolism in CeO_2_ NPs regulating Pb stress in rice.

## Figures and Tables

**Table 1:** Changes in photosynthetic pigment content of rice.

	Treatment	First Sampling	Second Sampling
	CK	39.142 ± 0.334 b	42.667 ± 1.504 a
Total chlorophyll content (mg·g^−1^)	Pb	41.628 ± 0.395 a	38.965 ± 0.494 b
	Ce50	41.740 ± 0.598 a	40.025 ± 0.413 ab
	CK	27.897 ± 0.176 b	29.086 ± 0.430 a
Chlorophyll a content (mg·g^−1^)	Pb	28.801 ± 0.176 a	27.794 ± 0.233 b
	Ce50	28.813 ± 0.181 a	28.197 ± 0.139 ab
	CK	11.245 ± 0.162 b	13.581 ± 1.094 a
Chlorophyll b content (mg·g^−1^)	Pb	12.827 ± 0.226 a	11.171 ± 0.270 b
	Ce50	12.928 ± 0.417 a	11.827 ± 0.277 ab
	CK	5.756 ± 0.026 b	5.975 ± 0.068 a
Carotenoid content (mg·g^−1^)	Pb	6.147 ± 0.058 a	5.739 ± 0.039 b
	Ce50	6.176 ± 0.022 a	5.938 ± 0.018 a

The different letters indicate significant differences (*p* < 0.05). The value represents the average ± standard error (three replicates).

## Data Availability

The data presented in this study are available on request from the corresponding author due to confidentiality.

## Supplementary Materials

The following supporting information can be downloaded at: https://www.mdpi.com/article/10.3390/antiox14050552/s1. Figure S1. KEGG annotation and TF gene statistics. AC, in the Pb/CK comparison group; BD, in the Ce50/Pb comparison group. Figure S2. Diterpenoid biosynthesis pathway in the Pb/CK comparison group. In the pathway diagram, red represents upregulated genes; blue represents downregulated genes. The copyright of the KEGG map has been obtained. Figure S3. Cluster analysis of DEGs in antioxidant activity and defense response in the Ce50/Pb comparison group. A, defense response; B, antioxidant activity. Figure S4. Differential metabolite classification. A, in the Pb/CK comparison group; B, in the Ce50/Pb comparison group. Figure S5. Differential metabolite abundance in the Pb/CK and Ce50/Pb comparison groups. Table S1. Primer sequence. Table S2. Sequencing data statistics. Table S3. Alignment result statistics. Table S4. Expression levels of stress-related genes
