# Supplementary material for: Foliar Spray of Cerium Oxide Nanoparticles (CeO2 NPs) Improves Lead (Pb) Resistance in Rice
Source: Antioxidants (Basel). 2025 May 7;14(5):552. doi: 10.3390/antiox14050552 (PMC12108155; doi:10.3390/antiox14050552)
Supplement: Supplementary file 1 [file antioxidants-14-00552-s001.zip › antioxidants-3528262-supplementary.pdf]

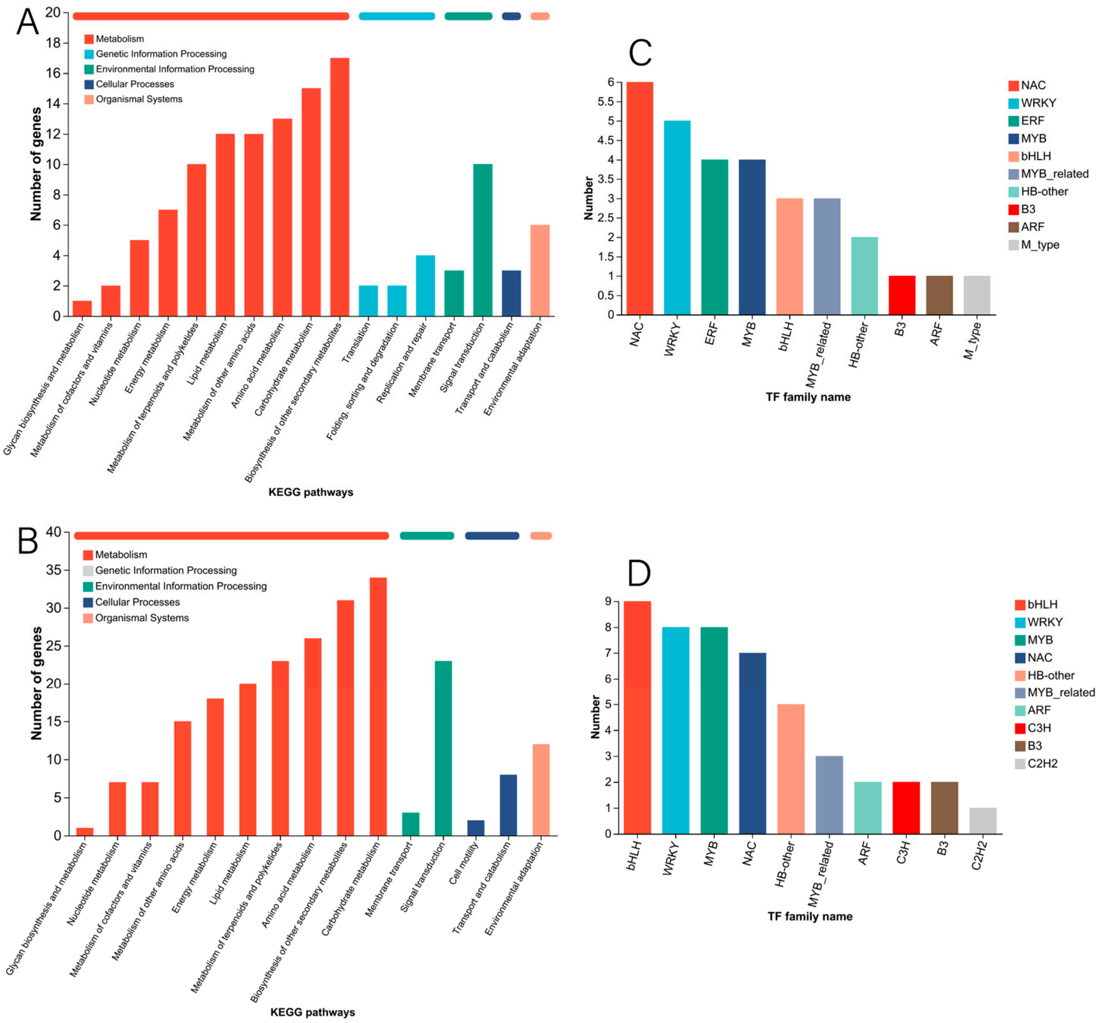

Figure S1. KEGG annotation and TF gene statistics. AC, in the Pb/CK comparison group; BD, in the Ce50/Pb comparison group.

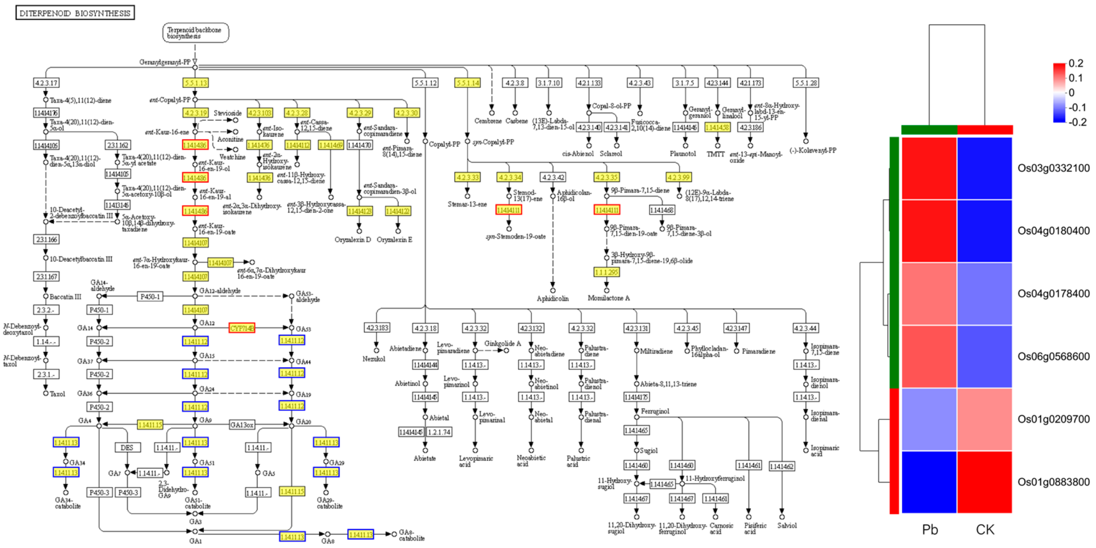

Figure S2. Diterpenoid biosynthesis pathway in the Pb/CK comparison group. In the pathway diagram, red represents upregulated genes; blue represents downregulated genes. The copyright of the KEGG map has been obtained.

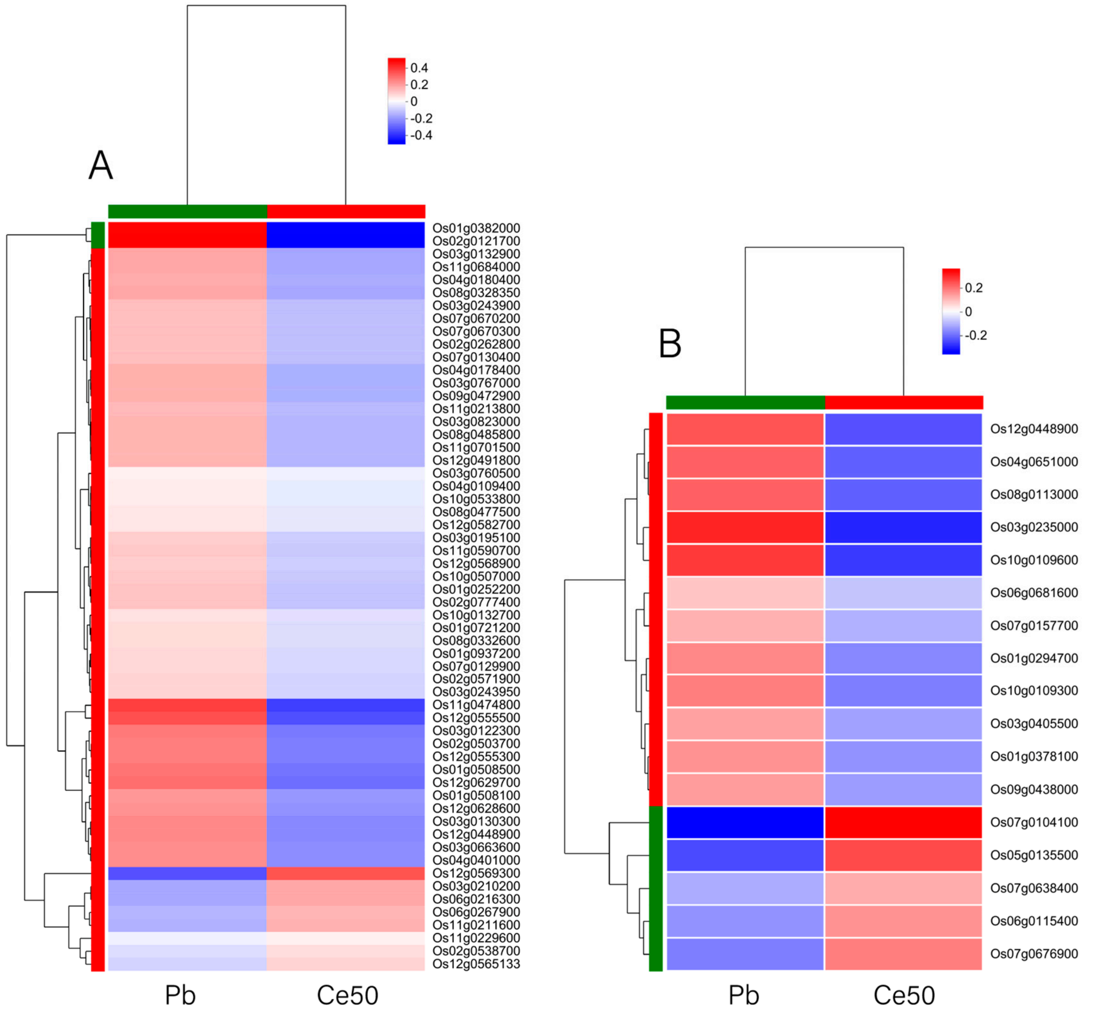

Figure S3. Cluster analysis of DEGs in antioxidant activity and defense response in the Ce50/Pb comparison group. A, defense response; B, antioxidant activity.

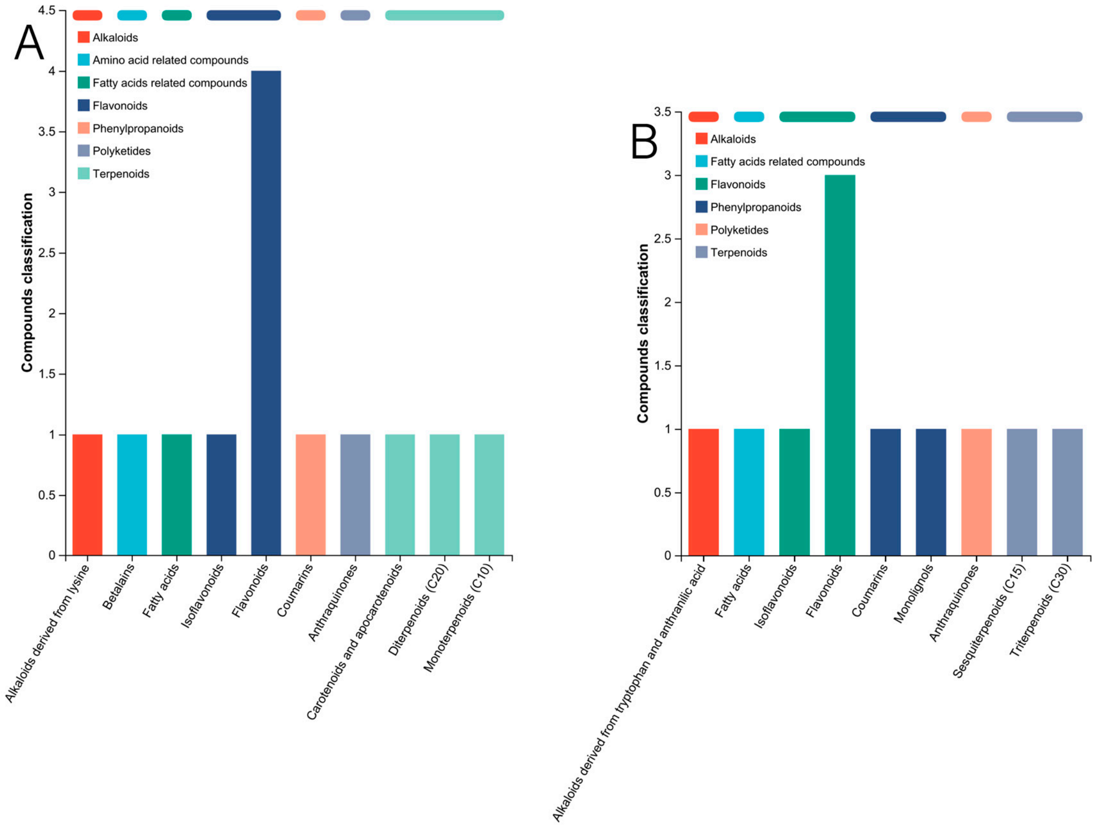

Figure S4. Differential metabolite classification. A, in the Pb/CK comparison group; B, in the Ce50/Pb comparison group.

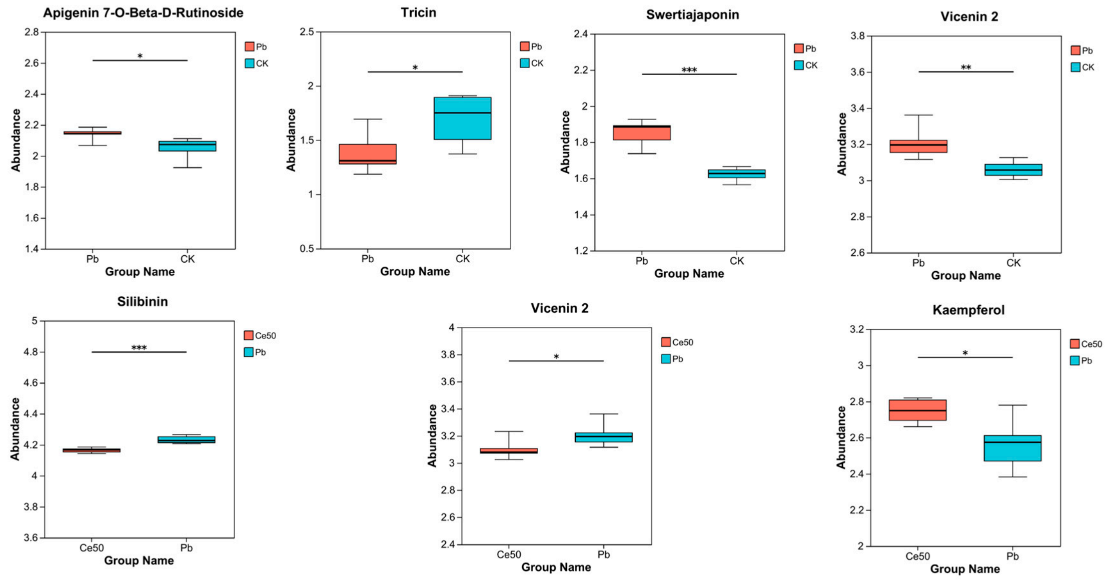

Figure S5. Differential metabolite abundance in the Pb/CK and Ce50/Pb comparison groups.

Table S1. Primer sequence

Gene ID		Primer sequence (5' to 3')
Os03g0183000	F	CTGTTCCAGCTCCCCTACTC
	R	GATTGGGAAGTCGTCGAAGC
Os03g0255100	F	CAAACTCTGTACCACGTGCC
	R	GATCACCACCAAACTGCTCA
Os03g0286900	F	TGGGTATTGAGTTCTGGATCGA
	R	CACCAGCACATACACCGC
Os04g0613700	F	TTTTGAGGTTTTGCGGGAGG
	R	ACTATGGAGACGAAGCAGCA
Os06g0115400	F	CTGCAGGGGAGAGAGTACG
	R	TCACAATGTCATCCTCGGTCA

Table S2. Sequencing data statistics

Sample	Raw reads	Raw bases	Clean reads	Clean bases	Error rate(%)	Q20(%)	Q30(%)	GC content(%)
CK(1)	40390654	6098988754	40200958	6037779927	0.0117	98.97	96.57	49.59
CK(2)	46998284	7096740884	46768774	7022761184	0.0117	98.94	96.47	48.92
CK(3)	45537592	6876176392	45334002	6806617711	0.0117	98.98	96.61	48.89
Pb(1)	45652482	6893524782	45441010	6823237079	0.0117	98.98	96.61	49.54
Pb(2)	50924880	7689656880	50689580	7613712506	0.0116	98.99	96.63	48.75
Pb(3)	39933056	6029891456	39739272	5973604692	0.0117	98.96	96.55	48.9
Ce50(1)	40738588	6151526788	40507464	6083057913	0.0117	98.96	96.54	49.28
Ce50(2)	46148926	6968487826	45909498	6895240102	0.0116	98.99	96.62	48.94
Ce50(3)	48107738	7264268438	47852776	7172573897	0.0117	98.94	96.49	49.54

Table S3. Alignment result statistics

Sample	Total reads	Total mapped	Multiple mapped	Uniquely mapped
CK(1)	40200958	37757901(93.92%)	3536194(8.8%)	34221707(85.13%)
CK(2)	46768774	43138031(92.24%)	3582938(7.66%)	39555093(84.58%)
CK(3)	45334002	42534120(93.82%)	2961521(6.53%)	39572599(87.29%)
Pb(1)	45441010	42617654(93.79%)	3855288(8.48%)	38762366(85.3%)
Pb(2)	50689580	47141373(93.0%)	2631409(5.19%)	44509964(87.81%)
Pb(3)	39739272	36865360(92.77%)	1949852(4.91%)	34915508(87.86%)
Ce50(1)	40507464	38077729(94.0%)	3318917(8.19%)	34758812(85.81%)
Ce50(2)	45909498	42896056(93.44%)	2745835(5.98%)	40150221(87.46%)
Ce50(3)	47852776	44822825(93.67%)	4151702(8.68%)	40671123(84.99%)

Table S4. Expression levels of stress-related genes

Gene ID	Gene Name	$2^{-(\Delta\Delta Ct)}$		TPM	
		Ce50	Pb	Ce50	Pb
Os03g0129400	*OsmiR528*			54.43	27.78
Os03g0183000	*OsLG3; OsERF62*	0.73	0.39	11.21	4.48
Os03g0255100		1.42	0.71	78.06	21.75
Os03g0286900	*OsRCI2-5*	1.24	0.37	15.36	1.23
Os04g0607500	*OsHKT1; 1*			15.71	6.26
Os04g0613700	*UAP2*	0.83	0.43	11.39	5.54
Os06g0115400	*SOD1-Fe*	1.86	1.2	61.31	30.12
Os12g0115700	*OsCHIL2*			87.04	41.90
